# Effect of *Spirulina platensis* Versus Simvastatin on the Skeletal Muscles of Experimentally Induced Dyslipidemia: A Multitarget Approach to Muscle Ultrastructural and Cytomolecular Modulation

**DOI:** 10.3390/medsci13030137

**Published:** 2025-08-15

**Authors:** Mai E. Abdelhady, Khaled H. Elmosalamy, Asmaa A. A. Kattaia, Mai A. Samak

**Affiliations:** 1 Department of Medical Histology and Cell Biology, Faculty of Medicine, Zagazig University, Zagazig 44519, Egypt; 2 College of Medicine, University of Ha’il, Ha’il 2240, Saudi Arabia

**Keywords:** *Spirulina platensis*, dyslipidemia, statin-induced myopathy, nutraceuticals, oxidative stress, TGF-β1, Bcl2

## Abstract

Background/Objectives: Dyslipidemia is a prevalent metabolic disorder closely linked to cardiovascular complications and muscular pathologies, often managed using statins such as simvastatin. However, statin-induced myopathy remains a significant treatment-limiting side effect, necessitating the exploration of safe, natural alternatives. *Spirulina platensis*, a phytochemical-rich marine-derived cyanobacterium, has emerged as a promising bioactive nutraceutical with potent antioxidant and anti-inflammatory properties. This study evaluated the comparative effects of *Spirulina platensis* and simvastatin in attenuating dyslipidemia-induced skeletal muscle injury in adult male albino rats. Methods: Forty animals were allocated to the control and high-fat diet (HFD) groups. After 4 weeks, the dyslipidemic rats were subdivided into untreated, simvastatin-treated, and *Spirulina platensis*-treated subgroups. Serum lipid profile, creatine kinase (CK), and malondialdehyde (MDA) levels were assessed. Histological, ultrastructural, and immunohistochemical analyses were conducted to assess muscle fiber integrity and expression of TGF-β1 and Bcl2. Results: *Spirulina platensis* significantly improved lipid parameters, reduced CK and MDA levels, preserved muscle histoarchitecture, and downregulated fibrotic (↓TGF-β1) and apoptotic (↑Bcl2) responses compared to the dyslipidemic and simvastatin-treated groups. Our results proved that *Spirulina platensis* ameliorates the effects of statin-associated myopathy while exerting lipid-lowering, cytoprotective, and antifibrotic effects. Conclusion: These molecular and ultrastructural benefits position *Spirulina platensis* as a promising, natural alternative to statins for managing dyslipidemia and preventing statin-induced myopathy. Future translational and clinical studies are warranted to further validate its efficacy and safety, supporting its broader application in metabolic and muscle-related disorders.

## 1. Introduction

Cardiovascular disease (CVD) remains the leading global cause of mortality, and dyslipidemia is recognized as a critical, modifiable metabolic contributor to its progression [1]. Defined by elevated serum triglycerides (TG), total cholesterol (TC), low-density lipoprotein cholesterol (LDL-C), and reduced high-density lipoprotein cholesterol (HDL-C), dyslipidemia is strongly associated with atherosclerosis, endothelial dysfunction, and reduced quality of life [2]. Accordingly, the development of effective lipid-lowering strategies is a central focus of modern research aimed at reducing the burden of atherosclerotic cardiovascular complications [3].

Statins, particularly simvastatin, are widely prescribed first-line agents for managing dyslipidemia and lowering cardiovascular risk [4]. Their lipid-lowering action is mediated through competitive inhibition of 3-hydroxy-3-methylglutaryl coenzyme A (HMG-CoA) reductase, the rate-limiting enzyme in endogenous cholesterol synthesis [5]. Despite their effectiveness, a considerable proportion of patients fail to reach target LDL-C levels, leaving them vulnerable to residual cardiovascular risk—especially those with familial hypercholesterolemia or multiple comorbidities [6].

Compounding this issue, simvastatin has been implicated in various degrees of statin-induced myopathy, ranging from myalgia and cramps to severe rhabdomyolysis characterized by elevated serum creatine kinase (CK) and risk of renal impairment [7]. These adverse muscular effects significantly restrict long-term statin adherence and efficacy, underscoring the urgent need for safer, well-tolerated alternatives with comparable lipid-lowering capabilities [8].

*Spirulina platensis*, a phytochemical-rich marine-derived blue-green microalga belonging to the Cyanobacteria phylum, thrives in alkaline and high-temperature aquatic environments [9]. More than 60% of its dry weight comprises high-quality protein, and it has a complete profile of essential amino acids, tannins, alkaloids, terpenoids, steroids, saponins, and phenolic acids. These phytochemicals are responsible for its medicinal and nutritional properties, and they occur naturally within the organism. Hence, Spirulina has earned recognition as a superfood [10]. One of its bioactive pigments, C-phycocyanin, has been shown to reduce serum TC and TG in preclinical studies [11]. Its phytochemical inventory’s hypoglycemic, antioxidant, anti-inflammatory, and immunomodulatory properties have positioned it as a promising candidate for nutraceutical and therapeutic applications [12].

In contrast to those of several other algae species, the safety profile of *Spirulina platensis* is well-established, with the U.S. Food and Drug Administration (FDA) granting it GRAS (Generally Recognized as Safe) status [13]. Toxicological studies have confirmed its non-toxic nature in both animal and human models [14]. Owing to its rich nutritional content and limited adverse effects, Spirulina has shown preventive and therapeutic impacts across a wide range of conditions, including fatty liver, diabetes, cardiovascular disease, and cancer [15]. Given the rising global burden of dyslipidemia and the increasing recognition of statin-associated muscle toxicity, there is a critical need for safer, bioactive alternatives that can manage lipid disorders without compromising skeletal muscle integrity. In this study, our goal was to determine whether *Spirulina platensis* offers a safer, marine-derived alternative to statins. By integrating biochemical, histological, ultrastructural, and immunohistochemical analyses, we sought to evaluate the extent to which *Spirulina platensis* can preserve muscle architecture and modulate key molecular markers of fibrosis and apoptosis, offering insights into its potential as a nutraceutical therapeutic intervention for lipid-related myopathies.

## 2. Materials and Methods

### 2.1. Chemicals

- Microalga *Spirulina platensis (Arthrospira platensis):* This marine-derived cyanobacterium was cultivated and obtained from the Microbiology Department, Faculty of Agriculture, Zagazig University. The chemical composition of the microalga was analyzed and confirmed at the Agriculture Research Center, Regional Center for Food and Feed. Afterwards, it was obtained as a refined dark blue green powder of ≥98% purity and stored far from light at room temperature for experimental use.
- Simvastatin: High-purity simvastatin ≥ 97% (HPLC, CAS No. 79902-63-9) was purchased from Sigma-Aldrich (Steinheim, Germany) for research purposes under agreement from Merck & Co., Inc., Kenilworth, NJ, USA. It was provided in the form of white powder and stored away from light at 2–8 °C.

### 2.2. Experimental Animals

Forty healthy adult male Wistar rats (10–14 weeks old, 250–270 gm) were used in this study. The rats were obtained and housed under specific pathogen-free conditions at the Breeding Animal House of the Faculty of Medicine, Zagazig University, Egypt. Prior to the experiment, the animals underwent a one-week acclimatization period to minimize stress-related variables. They were kept in separate stainless-steel cages, which were cleaned and sanitized regularly to maintain hygienic conditions, at a controlled temperature (23 ± 2 °C) and humidity (60 ± 5%) (12:12 h light–dark cycle; 8 a.m.–8 p.m.). Health was checked daily and body weights were recorded weekly by trained personnel to assess overall health, adjust dosages when necessary, and monitor the animals’ well-being throughout this study. All rats received humane care in compliance with the Ethical Committee of Zagazig University and in accordance with the NIH Guidelines for the Care and Use of Laboratory Animals (USA, No. 80–23, revised in 1996), approved by the Institutional Animal Care and Use Committee, Zagazig University (approval number: ZU-IACUC/3/F/59/2020).

### 2.3. Induction of Dyslipidemia

A total of 30 rats were fed a high-fat diet (HFD) prepared and obtained from the Nutrition and Clinical Nutrition department, Faculty of Veterinary Medicine, Zagazig University, Egypt, to induce hyperlipidemia. The HFD consisted of 60.5% standard rodent diet, 10% egg yolk powder, 17% beef tallow, 10% sucrose, 2% cholesterol, and 0.5% sodium cholate. This diet was specifically formulated to mimic the lipid-rich nutritional profile associated with dyslipidemia in humans. Lipid profile (TC, LDL, HDL, and TG) was estimated to confirm occurrence of dyslipidemia at the end of the 4th week, prior to further assignment of HFD rats into their subgroups. Additional hallmarks of dyslipidemia, such as oxidative overload markers and body weight indices, were utilized as conformational indicators.

### 2.4. Experimental Design

After acclimatization, the rats were randomly divided into two groups:

Group I (Control group)

Included 10 rats that were kept on a standard rodent diet for 8 weeks. These rats received no treatment.

Group II (High fat-diet group)

Included 30 rats that were fed on HFD for 8 weeks.

At the end of the 4th week, confirmed dyslipidemic rats were randomly subdivided into 3 subgroups:

- Subgroup IIa (Dyslipidemic group)

Included 10 rats that continued on the HFD for 4 weeks till the end of experiment without any treatment.

- Subgroup IIb (Simvastatin-treated group)

Included 10 rats that were kept on the HFD and received simvastatin via an orogastric tube at a dose of 80 mg/kg/day for 4 weeks. The simvastatin dose was calculated based on the human-equivalent dose using FDA guidelines for allometric scaling [16,17].

- Subgroup IIc (*Spirulina platensis*-treated group)

Included 10 rats that were kept on the high-fat diet and received *Spirulina platensis* at a dose of 500 mg/kg body weight daily via an orogastric tube for 4 weeks^.^ [18].

### 2.5. General Observations and Body Weight Measurements

During the experimental period, the general appearance and gait of rats and the amount of food intake were checked daily. No reported mortalities were recorded in the different groups.

Using a weighing scale (Hamar, made in China), the animals were weighed before study initiation (day 0) to determine the initial weights, at the end of 4th week to monitor weight gain, and at the end of the experiment to obtain the final weights. Then, the data were analyzed statistically.

### 2.6. Biochemical Analysis

- At the end of the experiment, all animals were fasted overnight; 2 mL of blood was collected in the morning from the orbital sinus with the help of a capillary tube by pressing the thumb behind the angle of the jaw, resulting in the engorgement of the retro-orbital plexus. The blood was centrifuged; serum was collected for estimation of lipid profile (TC, TG, LDL, and HDL) and serum creatine kinase (CK-MM) (as muscular damage injury). Lipid profile was measured twice: at the end of the 4th week (to confirm dyslipidemia in the HFD group) and at the end of the experiment by the 8th week. Dyslipidemia was confirmed by elevated concentration of TC (≥240 mg/dL), LDL-C (>160 mg/dL), and TGs (>200 mg/dL), along with reduced HDL-C (<40 mg/dL) [19].
- Determination of oxidative stress parameter, malondialdehyde (MDA), for monitoring lipid peroxidation rate in tissue samples [20]. Muscular samples were homogenized in 5–10 mL cold (4 °C) buffer (50 mM potassium phosphate at PH 7.5) and centrifuged (at 4000 rotations/min for 15 min), and the supernatant was submitted to a spectrophotometric assay (Rat malondialdehyde ELISA Kits, Bio Basic, Toronto, Canada). The results were expressed in nmol/g tissue.

### 2.7. Histological Study

At the end of the experiment, all rats were anesthetized by intraperitoneal injection of 200 mg/kg sodium pentobarbital solution [21]. The anesthetized rats were placed on a dissection board, and the belly of the gastrocnemius muscle was dissected away from the tendon to the insertion of the right limb of each rat and divided into small pieces. A piece of 1 cm^3^ from the mid-belly region of the muscle was obtained for histological processing. A piece of liver tissue was obtained for H&E staining to confirm the occurrence of hepatic steatosis [22].

#### 2.7.1. Light Microscope Study

The specimens of gastrocnemius muscles and liver tissue were fixed in 10% neutral buffered formalin, dehydrated, and then embedded in paraffin and processed for preparation of sections 5 µm thick. These sections were stained with

- *Hematoxylin and eosin stain (H&E)* for the gastrocnemius muscle and liver
- *Masson trichrome stain (MT)* for the gastrocnemius muscle.
- *Immuno-histochemical study*

Immunohistochemical reactions were carried out on paraffin sections of the gastrocnemius muscle for

A. Localization of Bcl2 protein: As an anti-apoptotic marker [23].
B. TGF-β1 immunostaining: As a pro-fibrotic marker [24].

Detection of Bcl2 protein and TGF-β1 protein were carried out using a streptavidin–biotin complex immunoperoxidase system. For detection of Bcl-2, the sections were incubated with rabbit monoclonal antibodies (Cat# MA5-16949, 1:300, R&D Systems, Santa Cruz Biotechnology, CA, USA). For detection of TGF-β1, the sections were incubated with rabbit monoclonal antibodies (CAS No. 85878, Sigma-Aldrich Chemical Company, St. Louis, MO, USA). Positive results for Bcl2 were indicated by brown cytoplasmic staining in muscle cells and connective tissue [25]. Positive results for TGF-β1 expression were indicated by brown cytoplasmic staining of the cells in the interstitial matrices [26].

#### 2.7.2. Transmission Electron Microscope Study

Sections of the gastrocnemius muscle were immediately fixed in 2.5% glutaraldehyde (pH 7.4). Then, they were post-fixed in 1% osmium tetroxide, dehydrated, and embedded in epoxy resin. Ultrathin sections (50 nm thick) were stained with double stain (uranyl acetate 5% followed by lead citrate). The prepared sections were examined and photographed using JEOL JEM 2100 electron microscope (Jeol Ltd., Tokyo, Japan) in the Electron Microscope unit of the Faculty of Agriculture, El Mansoura University, Egypt.

### 2.8. Morphometric Study

The image analyzer computer system (Image J soft) at the Image Analyzing Unit of the Anatomy Department, Faculty of Medicine, Zagazig University, was used to evaluate the area percentage of collagen, Bcl2 immune-reaction, and the number of TGF-β1 positive immune reactive cells. They were measured using the interactive measure menu. Ten readings from five non-overlapping sections from each rat of all groups were also examined.

### 2.9. Statistical Analysis

Data were analyzed using SPSS program (Statistical Package for Social Science) version 25.0. For normally distributed data, comparison between the studied groups was analyzed using t-test and one-way analysis of variance (ANOVA or F-test) and the least significant difference (LSD) test for pairwise comparisons. Given the limited and hypothesis-driven nature of the group comparisons, no correction for multiple testing was applied. A *p*-value < 0.05 was considered statistically significant, and this threshold was used consistently across all analyses. All data were expressed as mean + SD (standard deviation).

## 3. Results

### 3.1. Body Weight Results in Different Groups

Statistical analysis of the mean body weight using the t-test at the end of the 4th week revealed a highly statistically significant increase in body weight in the high fat-diet group compared to the control group (Table 1).

At the end of the experiment, LSD for comparison between groups showed a highly statistically significant increase in body weight in the dyslipidemic group compared to the other groups. There was a highly statistically significant decrease in body weight in the simvastatin- and *Spirulina platensis*-treated groups compared to the dyslipidemic group. On the other hand, there was no significant difference between the simvastatin- and *Spirulina platensis*-treated groups or between these groups and the control group (Table 2).

### 3.2. Biochemistry Results

#### 3.2.1. Serum CK (IU\L) Level

➢ There was a highly statistically significant increase in the mean serum CK in the skeletal muscle of the simvastatin-treated group and dyslipidemic group, respectively, compared to the other groups.
➢ A highly statistically significant decrease was detected in the *Spirulina platensis*-treated group.
➢ No statistically significant difference was revealed between the control and *Spirulina platensis*-treated groups (Table 3).

#### 3.2.2. Mean Value of Lipid Profile (mg/dL) Level After 4 Weeks

➢ There was a highly significant increase in the levels of TC, TG, and LDL in the high fat-diet group in comparison to the control group; however, the serum level of HDL was significantly decreased in comparison to the control group (Table 4).

#### 3.2.3. Mean Value of Lipid Profile (mg/dL) Level at End of Experiment

➢ There was a highly significant decrease in the levels of TC, TG, and LDL in the simvastatin-treated group and the *Spirulina platensis*-treated group in comparison to the dyslipidemic group. There was a highly significant increase in the serum level of HDL in the simvastatin-treated group and the *Spirulina platensis*-treated group compared to the dyslipidemic group. There was no significant difference between the simvastatin- and *Spirulina platensis*-treated groups (Table 5).

#### 3.2.4. Mean Value of Tissue MDA (nmol/g Tissue) Level

There was a highly statistically significant increase in the mean tissue MDA in the skeletal muscle of the simvastatin-treated group and dyslipidemic group, respectively, compared to the other groups. A highly statistically significant decrease was detected in subgroup IIc (*Spirulina platensis*-treated groups) compared to the simvastatin-treated group and the dyslipidemic group. No statistically significant difference was seen between the control and *Spirulina platensis*-treated groups (Table 6).

### 3.3. Light Microscopy Histological Results

#### 3.3.1. Hematoxylin and Eosin Stain (H&E) Results

**Figure 1:**
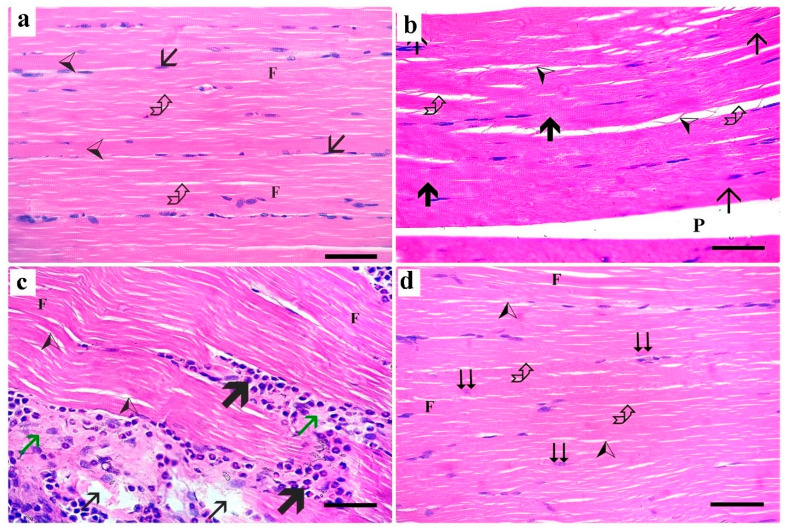
A photomicrograph of H&E-stained longitudinal section in the right gastrocnemius muscle. (**a**) Control group shows cylindrical elongated non-branched muscle fibers (F). The muscle fibers have acidophilic cytoplasm with transverse striations (arrowhead) and numerous flattened peripheral nuclei (arrow). Each muscle fiber is surrounded by a narrow endomysium (curved arrow). (**b**) Dyslipidemic group showing splitting (arrowhead) with increased endomysial spaces (curved arrow) and loss of striations (thin arrow). Some fibers with regular striations are also seen (thick arrow). Perimysium (P) is seen between skeletal muscle bundles. (**c**) simvastatin-treated group showing wavy muscle fibers with lost transverse striations (F), pale homogenous parts of some fibers (green arrow), splitting (arrow heads), areas of rarified cytoplasm (thin arrow) and sarcoplasmic cellular infiltration (thick arrow). (**d**) *Spirulina platensis*-treated group showing cylindrical muscle fibers (F) arranged in parallel with narrow endomysium in between (curved arrow). Some fibers with central vesicular nuclei are present (double arrow). Minimal splitting of muscle fibers is observed (arrowhead). (H&E, X 400, scale bar, 30 µm).

H&E-stained longitudinal sections (LSs) of the right gastrocnemius muscle of the control group showed normal skeletal muscle architecture. The muscle fibers appeared parallel, non-branching, and cylindrical in shape. Narrow endomysium was seen separating the skeletal muscle fibers. Each muscle fiber had acidophilic cytoplasm, with apparent transverse striations and numerous peripheral flat nuclei (Figure 1a). H&E-stained sections of the right gastrocnemius muscle of the dyslipidemic group revealed disruption of skeletal muscle fibers with loss of striations and splitting in the affected regions (Figure 1b). H&E-stained sections of the right gastrocnemius muscle of the simvastatin-treated group revealed complete loss of transverse striations and more splitting of myofibrils. There was massive destruction with dramatic loss of muscle architecture, with pale homogenous parts of some fibers and sarcoplasmic cellular infiltration. Loss of cytoplasmic structure with areas of rarified tissue and vacuolation were found with cellular infiltration and blood vessel congestion (Figure 1c). The H&E-stained longitudinal section in the right gastrocnemius muscle of the *Spirulina platensis* group showed apparently normal skeletal muscle architecture. Longitudinal sections showed that most of the muscle fibers appeared normal, with peripheral flat nuclei and cross striations. Some muscle fibers with central vesicular nuclei were noticed. Narrow endomysial spaces were also seen. However, splitting of some fibers was still observed (Figure 1d).

**Figure 2:**
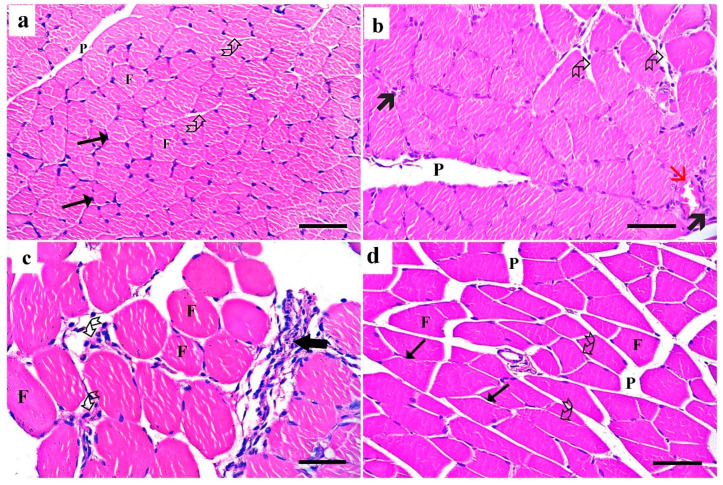
A photomicrograph of H&E-stained transverse sections of gastrocnemius muscle. (**a**) Control group shows polyhedral muscle fibers (F) with peripheral nuclei (arrow) and acidophilic cytoplasm that are separated by narrow endomysium (curved arrow). The perimysium (P) is seen between muscle bundles. (**b**) Dyslipidemic group showing increased endomysial spaces (curved arrow), some inflammatory cell infiltrate (thick arrow), and congested blood vessel (red arrow). Note the perimysium (P) between skeletal muscle bundles. (**c**) Simvastatin-treated group showing variably sized muscle fibers exhibiting acidophilic sarcoplasm with nonclear myofibrils (F). Wide endomysial spaces are present between the muscle fibers with cellular infiltration (curved arrow). Note cellular infiltration also (thick arrow) in the perimysium. (**d**) *Spirulina platensis*-treated group showing polyhedral muscle fibers (F) with peripheral nuclei (arrow) and acidophilic cytoplasm separated by narrow endomysium (curved arrow). Note the perimysium (P) between muscle bundles. (H&E, X 400, scale bar, 30 µm).

Examination of the H&E-stained transverse sections in the right gastrocnemius muscle of the control group showed polyhedral muscle fibers with peripheral nuclei and acidophilic cytoplasm separated by narrow endomysium (Figure 2a). The dyslipidemic group showed a congested blood vessel, some inflammatory cellular infiltrate, and wide endomysial spaces (Figure 2b). In the simvastatin-treated group, the muscle fibers were of variable sizes and exhibited pale homogenous acidophilic sarcoplasm with nonclear myofibrils and cellular infiltration in the endomysium and perimysium (Figure 2c). The transverse section of the gastrocnemius muscle of the *Spirulina platensis*-treated group showed almost normal polyhedral muscle fibers with peripheral nuclei separated by narrow endomysium (Figure 2d).

**Figure 3:**
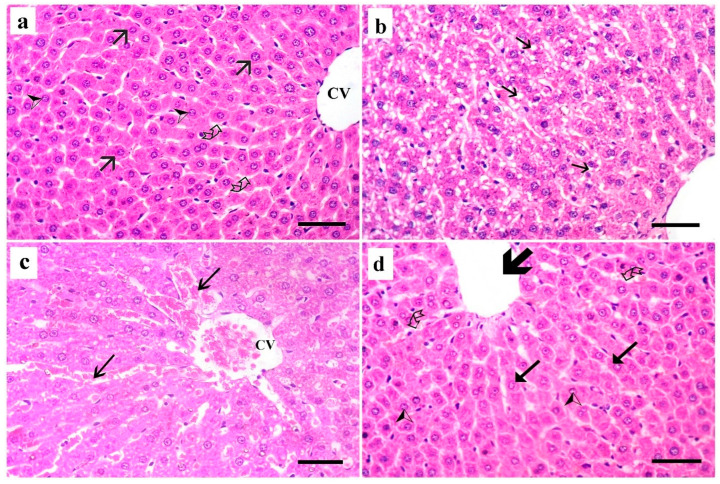
H&E-stained section of liver tissue. (**a**) Control group shows normal hepatocytes (arrow) with vesicular nuclei (arrowhead) around the normal central vein (CV). Blood sinusoids (curved arrow) appear normal, separating hepatocyte cords. (**b**) Dyslipidemic group shows most hepatocytes with vacuolated cytoplasm (arrow). (**c**) Simvastatin-treated rats showing congestion of central vein (CV) and blood sinusoids (arrow). (**d**) *Spirulina platensis*-treated group shows almost normal hepatic architecture with normal hepatocytes that are polyhedral in shape (arrow) and exhibit normal arrangements and central vesicular nuclei (arrowhead). Mild congested blood sinusoids (curved arrow) and average-sized central vein (thick arrow) are seen. (H&E, X 400, scale bar, 30 µm).

Examination of the H&E-stained section of liver tissue of the control group showed normal histological structure of the hepatic tissue formed of normal central veins and hepatocytes cords radiating from them. The hepatocytes appeared polyhedral, with vesicular nuclei. Blood sinusoids appeared normal, separating hepatocyte cords (Figure 3a). A section of the dyslipidemic group showed that most hepatocytes exhibited vacuolated cytoplasm. The blood sinusoids appeared slightly dilated (Figure 3b). The simvastatin-treated group showed congestion of the central vein and blood sinusoids (Figure 3c). The *Spirulina platensis* group showed normal hepatocytes polyhedral in shape with central vesicular nuclei with normal arrangements and architecture with mild dilated blood sinusoids (Figure 3d).

#### 3.3.2. Masson Trichrome Stain (MT) Results

**Figure 4:**
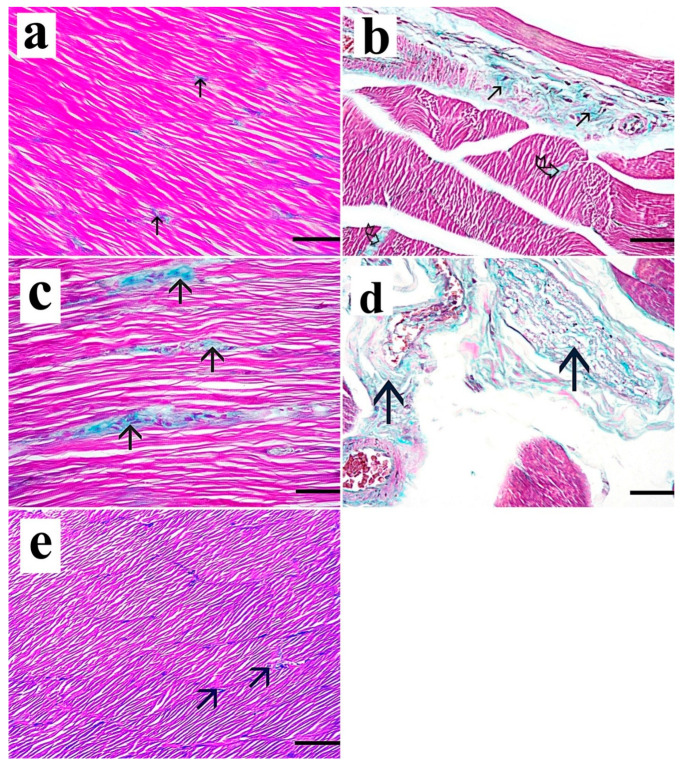
Masson trichrome-stained sections of the right gastrocnemius muscle. (**a**) Control group shows few collagen fibers between muscle fibers (arrow). (**b**) Dyslipidemic group shows a moderate increase in the collagen fibers in the perimysium (arrow) and also in the endomysium (curved arrow). (**c**,**d**) Simvastatin-treated group shows marked increase in the collagen fibers between the muscle fibers (arrow) and in the collagen fibers in the wide perimysium between the muscle bundles (arrow). (**e**) *Spirulina platensis*-treated group shows a few collagen fibers between muscle fibers (arrow). (Masson trichrome, X 400, scale bar, 30 µm).

Masson trichrome-stained sections of the right gastrocnemius muscle of the control group and the *Spirulina platensis* group revealed few collagen fibers between muscle fibers (Figure 4a,e). Sections of the dyslipidemic group revealed a moderate increase in the collagen fibers between the muscle fibers and in the perimysium (Figure 4b). The simvastatin-treated group showed a marked increase in the collagen fibers between the muscle fibers as well as in the wide degenerated areas (Figure 4c,d).

#### 3.3.3. Immunohistochemical Results of Pro-Fibrotic and Anti-Apoptotic Markers

**Figure 5:**
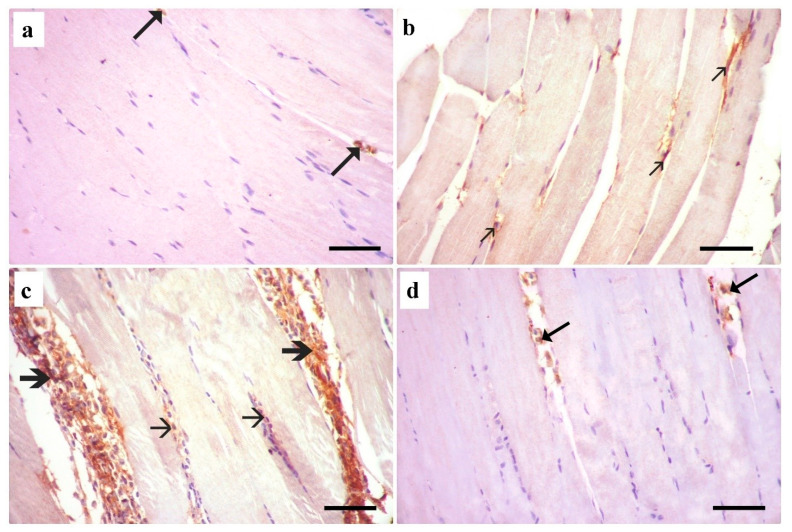
Immunohistochemical stained gastrocnemius muscle longitudinal sections for anti-TGFβ1. (**a**) Control group shows scanty brownish immunostained cells in the endomysium (arrow) between skeletal muscle fibers. (**b**) Dyslipidemic group shows some brownish immunostaining in the endomysium (arrow) between skeletal muscle fibers. (**c**) Simvastatin-treated group showing excessive brownish cytoplasmic immunostaining in the cells in the endomysium (thin arrow) and perimysium (thick arrow). (**d**) *Spirulina platensis*-treated group shows a few brown immunostained cells (arrow) in the endomysium between skeletal muscle fibers. (Immunoperoxidase technique for TGFβ1, X 400, scale bar, 30 µm).

Immunohistochemical stained longitudinal sections for TGFβ1 (pro-fibrotic) of the control group showed scanty brownish cytoplasmic immunostaining in the endomysium between skeletal muscle fibers (Figure 5a). Longitudinal sections for TGFβ1 of the dyslipidemic group showed some brownish cytoplasmic immunostaining in the endomysium between skeletal muscle fibers (Figure 5b). Excessive brownish cytoplasmic immunostaining in the endomysium between skeletal muscle fibers appeared in the simvastatin-treated group (Figure 5c). Minimal brownish cytoplasmic immunostaining in the endomysium between skeletal muscle fibers appeared in the *Spirulina platensis*-treated rats (Figure 5d).

**Figure 6:**
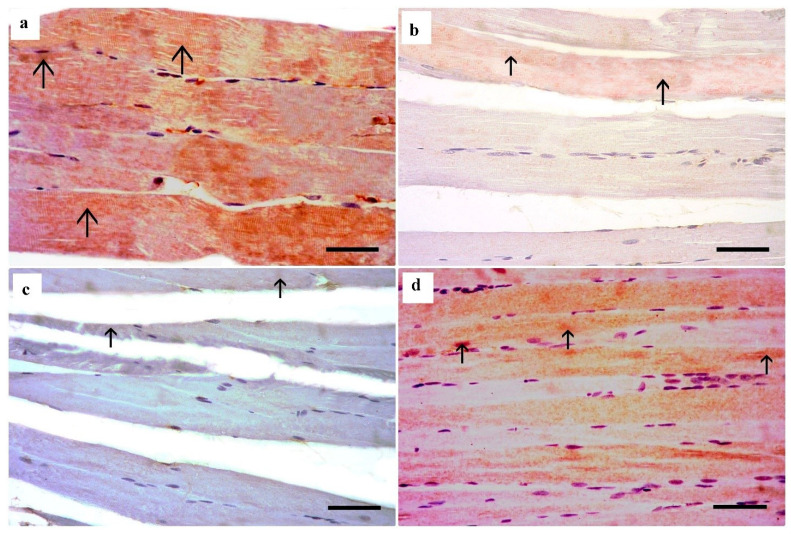
Immunohistochemical reaction for anti-Bcl2 in gastrocnemius muscle longitudinal sections. (**a**) Control group shows strong positive Bcl2 immunoreactivity, demonstrated by diffuse cytoplasmic staining in the myocytes (arrow). (**b**) Dyslipidemic group reveals mild positive immunoreactivity, demonstrated by some brownish cytoplasmic staining (arrow) in the skeletal muscle fibers. (**c**) Simvastatin-treated group reveals minimal brownish cytoplasmic staining in the myocytes (arrow). (**d**) *Spirulina platensis*-treated group showing excessive brown cytoplasmic staining (arrow) in the skeletal muscle fibers. (Immunoperoxidase technique for Bcl2, X 400, scale bar, 30 µm).

Immunohistochemical reaction for Bcl2 in longitudinal sections of the control rats revealed strong positive Bcl2 immunoreactivity, demonstrated by diffuse cytoplasmic staining in the myocytes (Figure 6a). The dyslipidemic rats revealed mild positive immunoreactivity, demonstrated by some brownish cytoplasmic staining in the myocytes (Figure 6b). The simvastatin-treated group revealed minimal cytoplasmic immunoreactivity, demonstrated by faint brownish cytoplasmic staining in the myocytes (Figure 6c). Immunohistochemical reaction for Bcl2 in longitudinal sections of the *Spirulina platensis*-treated rats revealed positive immunoreactivity, demonstrated by excessive cytoplasmic staining in the myocytes (Figure 6d).

### 3.4. Transmission Electron Microscopy Results

**Figure 7:**
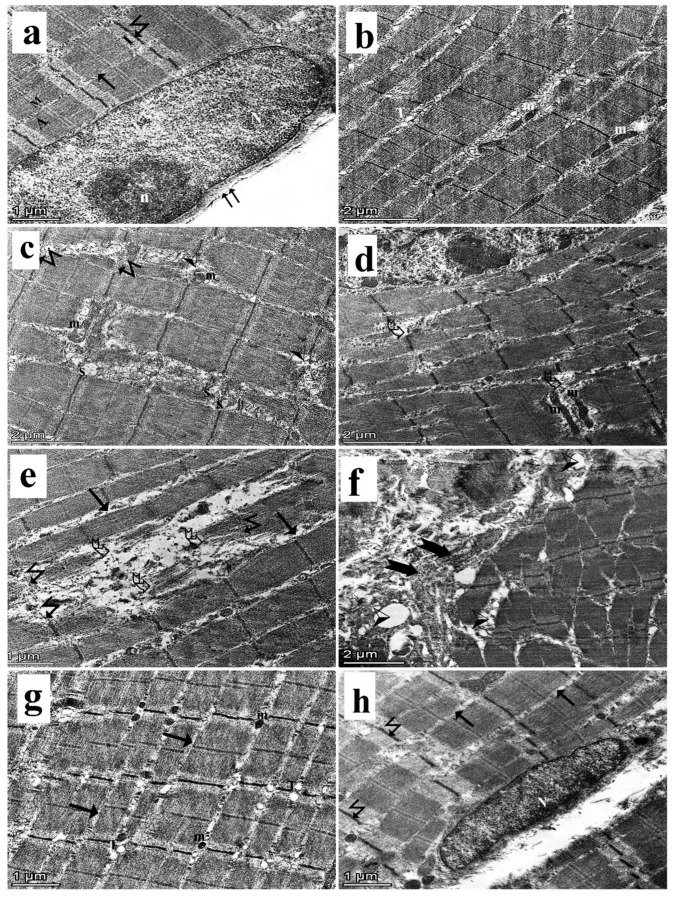
TEM of the gastrocnemius muscle ultrathin sections. (**a**,**b**): Control group shows parallel arrangement of the myofibrils with narrow intermyofibrillar space (arrow). Z-line (zigzag arrow) is seen bisecting the light band (I). A dark electron-dense line (M line) is seen at the center of the dark band (A). Note that the triad at the A-I junction (T) and mitochondria in the intermyofibrillar space (m), along with a euchromatic nucleus (N) with a prominent nucleolus (n), are present just beneath the sarcolemma (double arrow). (**c**,**d**) Dyslipidemic group shows interrupted Z-line (zigzag arrow), irregular mitochondria with ruptured cristae (m), dilated SER (S), and wide spaces (arrowhead). Degenerated myofibrils (curved arrow) and nucleus (N) with irregular outline are observed. (**e**,**f**) Simvastatin-treated group shows degenerated myofibrils with areas of rarified cytoplasm (curved arrow), increased intermyofibrillar spaces (arrow), and interrupted Z-line (zigzag arrow). Accumulation of collagen fibers (bifid arrow) and many spaces (arrowhead) in the connective tissue between muscle fibers are also observed. (**g**,**h**) *Spirulina platensis*-treated group shows regular arrangement of the myofibrils with euchromatic nucleus (N), narrow intermyofibrillar space (arrow), and regular Z-line (zigzag arrow). A triad is observed at the A-I junction (T), and mitochondria are also seen in intermyofibrillar spaces (m) (TEM, X 22,000, scale bar, 2 µm; TEM, X 17,000, scale bar, 1 µm).

Examination of the ultrathin sections of the gastrocnemius muscles of the control group showed the normal arrangement of skeletal muscle fibers with narrow intermyofibrillar spaces. Regular architecture of alternating light (I) and dark (A) bands was observed. At the middle of the light band, a Z-line was seen. An electron-lucent narrow region, the H band, was seen at the center of the A band, with a dark electron-dense line (M line) within it. The euchromatic nucleus was found peripherally just under the sarcolemma, with a prominent nucleolus. Some mitochondria were seen in the intermyofibrillar space. Triad was observed at the A-I junction (Figure 7a,b). The dyslipidemic group showed disruption of the normal architecture of the skeletal muscular tissue in the form of degenerated parts of myofibrils with an interrupted Z-line. Mitochondria with ruptured cristae, irregularly shaped mitochondria, dilated SER, wide spaces, and nuclei with irregular outlines were also detected (Figure 7c,d). Ultrathin sections of the simvastatin-treated group showed loss of the normal architecture of the skeletal muscular tissue in the form of focal degeneration and fragmentation of the myofibrils, areas of rarified cytoplasm, interrupted Z-lines, and increased intermyofibrillar spaces (Figure 7e). Some fibers in the same group showed accumulation of collagen fibers and many spaces in the connective tissue between muscle fibers (Figure 7f). The *Spirulina platensis* group showed a relatively normal pattern and arrangement of myofibrils with light and dark bands, clear successive Z-lines (Z), and decreased intermyofibrillar spaces containing mitochondria (Figure 7g,h).

### 3.5. Morphometrical Results

Statistical analysis of the area % for collagen fibers’ morphometrical results using a one-way ANOVA test revealed a highly statistically significant increase in the mean area % for collagen fibers in subgroups IIb and IIa (simvastatin-treated group and dyslipidemic group) (8.89 ± 2.81, 1.45 ± 0.46), respectively, compared to the other groups. In addition, there was a highly statistically significant decrease in the mean area % for collagen fibers in the *Spirulina platensis*-treated group (1.09 ± 0.34.) However, there was no statistically significant difference between the control (0.66 ± 0.21) and *Spirulina platensis*-treated groups (Table 7).

Statistical analysis of the area % for Bcl2 immunoreaction morphometrical results using a one-way ANOVA test revealed a highly statistically significant difference among the different groups studied, as the *p* value < 0.001. LSD for comparison between the groups revealed a highly statistically significant decrease in the mean area % for Bcl2 reaction in the skeletal muscle of the simvastatin-treated group and the dyslipidemic group (4.99 ± 1.45, 18.32 ± 4.1), respectively, compared to the other groups. A highly statistically significant increase was observed in the *Spirulina platensis*-treated group compared to the simvastatin-treated group and the dyslipidemic group. However, there was no statistically significant difference between the control group (41.14 ± 4.26) and the *Spirulina platensis*-treated group (38.95 ± 2.32) (Table 8).

Statistical analysis of the morphometrical results for the number of TGFβ1 positive cells using a one-way ANOVA test showed a highly statistically significant difference among the different groups studied, as the *p* value was < 0.001. LSD for comparison between the groups revealed a statistically significant increase in the number of TGFβ1 positive cells in the skeletal muscle of the dyslipidemic group (22.00 ± 2.404) and a highly statistically significant increase in the number of TGFβ1 positive cells in the skeletal muscle of the simvastatin-treated group (133.70 ± 30.862). A highly statistically significant decrease was observed in subgroup IIc (*Spirulina platensis*-treated group) compared to the simvastatin-treated group. However, there was no statistically significant difference between the control group (4.60 ± 0.516) and the *Spirulina platensis*-treated group (7.20 ± 1.751) (Table 9).

## 4. Discussion

Statins, such as simvastatin, are widely prescribed as first-line hypolipidemic agents for managing hypercholesterolemia and mixed dyslipidemia due to their proven cardiovascular benefits [6]. However, their clinical use is limited by the development of statin-associated muscle symptoms [27]. As an alternative, *Spirulina platensis*, a phytochemical-rich cyanobacterium, has gained attention for its diverse pharmacological effects, including hypolipidemic, anti-inflammatory, and antioxidant activities, which may be beneficial in treating dyslipidemia-induced muscle damage [28]. We adopted the *Spirulina platensis* dose of 500 mg/kg/day for our study in alignment with the upper safety threshold in rodent studies, which is equivalent to a human dose of approximately 5.6 g/day for a 70 kg adult, according to basic allometric scaling based on body surface area [18,29].

By the end of the fourth week of our study, rats fed a high-fat diet (HFD) exhibited significant weight gain and biochemical signs of metabolic stress, consistent with previous models of obesity and insulin resistance, confirming effective induction of obesity and dyslipidemia [30]. This finding aligns with previous reports indicating that HFD promotes adipose tissue expansion, insulin resistance, and hepatic fat accumulation [31,32]. Increased free fatty acid levels and metabolic disturbances associated with an HFD can impair PKB/Akt signaling, downregulate GLUT-4, and promote glycogenesis, thereby contributing to body mass gain [33]. Additionally, both Spirulina and simvastatin treatment significantly mitigated HFD-induced weight gain, consistent with the prior finding that simvastatin regulates lipid metabolism [31], while Spirulina exerts anti-obesity effects via C-phycocyanin and unsaturated fatty acids [34]. These findings are in agreement with prior reports of Spirulina’s metabolic regulatory actions, including the modulation of lipid metabolism, appetite control, and energy homeostasis [35,36].

We evaluated muscle damage via creatine kinase and malondialdehyde levels; both the HFD and simvastatin-treated groups showed a marked increase in serum CK levels, suggesting significant skeletal muscle damage. This is in line with reports of CK elevation due to HFD-induced muscle dysfunction [37,38], likely resulting from altered energy metabolism and oxidative stress [39]. Notably, the *Spirulina platensis*-treated group showed a significant reduction in CK, reflecting better muscle preservation. The observed increase in CK levels in the group treated with simvastatin supports its known myotoxic profile, consistent with the findings of Choi et al. (2016) and Ghalwash et al. (2018) [40,41]. While Piette et al. (2016) [42] reported no significant myopathy with short-term statin use, others have attributed statin-induced CK leakage to mitochondrial dysfunction, cell membrane instability, and apoptotic signaling pathways [43,44]. In contrast, our findings revealed that *Spirulina platensis* supplementation significantly lowers serum CK, likely due to its antioxidant properties and ability to preserve muscle fiber integrity [45]. This is consistent with studies showing reduced CK and LDH levels in athletes following Spirulina intake [46]. The presence of branched-chain amino acids in this microalga may provide metabolic support, reduce protein catabolism, and enhance muscle repair [47,48]. Fat oxidation promotion, via increased mitochondrial efficiency or upregulated β-oxidation pathways, may help mitigate metabolic stress and reduce muscle fiber damage, thereby contributing to lower serum CK levels [49].

In the biochemical domain, the dyslipidemic group exhibited significantly elevated TC, TG, and LDL levels and reduced HDL, reflecting classical HFD-induced hyperlipidemia [50]. Meanwhile, both simvastatin and *Spirulina platensis* significantly improved lipid profiles, lowering TC, TG, and LDL and increasing HDL. Simvastatin acts via HMG-CoA reductase inhibition to reduce endogenous cholesterol synthesis and hepatic lipid accumulation [51]. *Spirulina platensis* also showed strong hypolipidemic effects, attributed to its content of polyunsaturated fatty acids and antioxidants, such as phycocyanins and phenolics [52,53]. Gamma-linolenic acid in Spirulina enhances cholesterol esterification, while its low-calorie protein content and stimulation of hepatic lipase activity promote a healthier lipid profile [54]. The injurious effect of dyslipidemia owing to increased MDA levels in the gastrocnemius muscle of dyslipidemic and simvastatin-treated rats confirmed the presence of lipid peroxidation and oxidative stress. Simvastatin-induced oxidative damage has been previously documented [16]; on the other hand, Spirulina’s ability to decrease MDA levels and enhance antioxidant defenses further supports its cytoprotective role [55].

Histopathologically, dyslipidemic muscles showed fiber disruption, loss of striations, and collagen accumulation—hallmarks of muscle degeneration and fibrosis driven by oxidative damage and inflammation [56]. These changes were linked to elevated TGF-β1 expression, a major profibrotic cytokine [57]. Simvastatin administration further aggravated these structural defects, as revealed by observable myofiber destruction, vacuolization, and collagen deposition—findings aligned with those of Kwak (2014) and Chogtu et al. (2020) [58,59]. Mechanistically, statin-induced myopathy may result from mitochondrial CoQ10 deficiency, ROS generation, Ca^2+^ imbalance, and apoptosis [60,61]. Furthermore, the marked reduction in Bcl2 expression in this group additionally supports mitochondria-mediated apoptosis [62]. Notably, Masson trichrome staining and EM analysis confirmed pronounced fibrosis in the simvastatin group, with dense collagen replacing degenerated fibers. TGF-β1 upregulation likely triggered this fibrotic cascade by activating myofibroblasts and promoting ECM deposition [63]. In contrast, *Spirulina platensis* significantly preserved muscle architecture, with minimal collagen deposition. Reduced TGF-β1 levels may explain the limited fibrosis, consistent with previous reports highlighting Spirulina’s antifibrotic activity via TGF-β1/α-SMA modulation [64].

The increased Bcl2 expression observed in the Spirulina-treated group supports its anti-apoptotic role, consistent with Afkhami-Ardakani et al. (2017), who demonstrated Bcl2 upregulation in testicular tissue following Spirulina treatment [65]. Minor signs of myofiber splitting were noted, indicating active regeneration. Moreover, hypercellularity and central nuclei reflect regenerative phases in muscle healing [66]. Spirulina’s known antioxidant and anti-inflammatory properties likely supported this process, as previously reported [67].

Spirulina’s therapeutic potential is further supported by its high content of C-phycocyanin and β-carotene, which suppress oxidative and inflammatory mediators, such as iNOS, COX-2, TNF-α, and NF-κB [68,69]. Additionally, C-phycocyanin may activate Nrf2, a key regulator of antioxidant defense, providing cytoprotection against oxidative stress [70,71]. Consequently, Nrf2 activation through *Spirulina* can be considered a form of cytoprotection, as it helps cells adapt to and defend themselves against various stressors, including oxidative stress, toxic compounds, and inflammation. In summary, this study provides robust evidence that *Spirulina platensis*, enriched with diverse phytochemicals such as phycocyanin, polyunsaturated fatty acids, and phenolics, offers a multitarget therapeutic approach to dyslipidemia-induced muscle injury and statin-associated myopathy. Spirulina not only improves lipid profiles, reducing total cholesterol, LDL, and triglycerides while increasing HDL, but also exhibits potent antioxidant and anti-inflammatory activities, thereby mitigating oxidative stress and preserving muscle ultrastructure. Notably, *Spirulina platensis* administration significantly attenuated fibrotic (↓TGF-β1) and apoptotic (↑Bcl2) responses in skeletal muscle, highlighting its cytoprotective and antifibrotic potential. Although both agents exhibited comparable lipid-lowering effects, *Spirulina platensis* demonstrated superior protective effects in skeletal muscle architecture, oxidative stress modulation, and cytomolecular markers compared to simvastatin. These molecular and ultrastructural benefits position *Spirulina platensis* as a promising, natural alternative to statins for managing dyslipidemia and preventing statin-induced myopathy. Future translational and clinical studies are warranted to further validate its broader application in metabolic and muscle-related disorders.

Limitations of the study:

Addressing these limitations is warranted to strengthen the translational applicability of *Spirulina platensis* as a therapeutic alternative to conventional statins. First, the relatively short duration of treatment may not adequately reflect the long-term efficacy or safety profile of either intervention. Second, simvastatin- and Spirulina-treated groups were included under normolipidemic conditions to isolate the direct effects of each treatment. Third, the lack of functional assessments of muscle performance (e.g., grip strength, fatigue resistance) limits interpretation of the physiological relevance of structural improvements. Fourth, while molecular markers such as Bcl2 and TGF-β1 were evaluated, a more comprehensive pathway analysis involving upstream or downstream regulators would provide deeper mechanistic insights.

## Figures and Tables

**Table 1:** Comparing body weight in the studied groups at the end of 4th week using *t*-test.

Group	(Control Group) (n = 10)	(High Fat-Diet Group) (n = 30)	T	*p* Value
	Mean ± SD of Body Weight at the End of 4th Week	Mean ± SD of Body Weight at the End of 4th Week		
4th Week	263.50 ± 5.798	306.50 ± 19.305	−6.886	<0.001 **

Data were expressed as mean ± SD Independent sample *t* test ** highly significant (*p* ≤ 0.001).

**Table 2:** Comparing body weight in the studied groups at the end of the experiment using ANOVA test.

Group	(Control Group)	(Dyslipidemic Group)	(Simvastatin-Treated Group)	(*Spirulina platensis*-Treated Group)	F	*p* Value	LSD
	Mean ± SD	Mean ± SD of Body Weight at the End of Experiment	Mean ± SD of Body Weight at the End of Experiment	Mean ± SD of Body Weight at the End of Experiment			
8th week (End of experiment)	272.50 ± 7.169	486.00 ± 56.016	276.00 ± 6.583	280.40 ± 7.074	134.091	<0.001 **	P1 < 0.001 ** P2 = 0.786 P3 = 0.541 P4 < 0.001 ** P5 < 0.001 ** P6 = 0.733

Data are expressed as mean ± SD. ANOVA test (analysis of variance), LSD (least significant difference; ** highly significant (*p* ≤ 0.001); P1 = control group vs. dyslipidemic group; P2 = control group vs. simvastatin-treated group; P3 = control group vs. *Spirulina platensis*-treated group; P4 = dyslipidemic group vs. simvastatin-treated group; P5 = dyslipidemic group vs. *Spirulina platensis*-treated group; P6 = simvastatin-treated group vs. *Spirulina platensis*-treated group.

**Table 3:** Comparing serum CK IU/L in the studied groups using ANOVA test.

Group	(Control Group)	(Dyslipidemia Group)	(Simvastatin-Treated Group)	(*Spirulina platensis*-Treated Group)	F	*p* Value
	Mean ± SD of **Serum CK**	Mean ± SD of **Serum CK**	Mean ± SD of **Serum CK**	Mean ± SD of **Serum CK**		
LSD	327.7 ± 52.7	439.5 ± 20.47	589.0 ± 70.15	375.0 ± 44.17	54.4	<0.001 **
	(Control group)	P1 <0.001 **	P2 <0.001 **	P3 = 0.2		
	(Dyslipidemia group)			P4 < 0.001 **		
	(Simvastatin-treated group)			P5 < 0.001 **		

Data were expressed as mean ± SD; One-way ANOVA test (analysis of variance); LSD (least significant difference); ** highly significant (*p* ≤ 0.001); P1 = control group vs. dyslipidemic group; P2 = control group vs. simvastatin-treated group; P3 = control group vs. *Spirulina platensis*-treated group; P4 = dyslipidemic group vs. *Spirulina platensis*-treated group; P5 = simvastatin-treated group vs. *Spirulina platensis*-treated group.

**Table 4:** Comparing serum lipid profile in the studied groups after 4 weeks using *t*-test.

Group	(Control Group) (n = 10)	High Fat-Diet Group (n = 30)	T	*p* Value
	Mean ± SD of **Serum Lipid Profile** After 4 Weeks	Mean ± SD of **Serum Lipid Profile** After 4 Weeks		
Total cholesterol	98.27 ± 7.87	269.5 ± 24.89	−21.2	<0.001 **
Triglyceride	92.78 ± 6.79	253.90 ± 23.87	−20.8	<0.001 **
LDL	92.30 ± 9.34	234.50 ± 18.25	−23.4	<0.001 **
HDL	56.60 ± 7.84	12.96 ± 4.16	22.65	<0.001 **

Data are expressed as mean ± SD; Independent sample *t*-test; ** highly significant (*p* ≤ 0.001).

**Table 5:** Comparing serum lipid profile in the studied groups at the end of the experiment using ANOVA test.

Group	(Control Group)	(Dyslipidemic Group)	(Simvastatin-Treated Group)	(*Spirulina platensis*-Treated Group)	F	*p* Value	LSD
	Mean ± SD of **Serum Lipid Profile** at End of Experiment	Mean ± SD of **Serum Lipid Profile** at End of Experiment	Mean ± SD of **Serum Lipid Profile** at End of Experiment	Mean ± SD of **Serum Lipid Profile** at End of Experiment			
Total cholesterol	96.60 ± 8.93	295.00 ± 21.60	162.60 ± 7.53	137.40 ± 5.60	464.27	<0.001 **	P1 < 0.001 ** P2 < 0.001 ** P3 < 0.001 ** P4 < 0.001 ** P5 < 0.001 ** P6 < 0.001 **
Triglyceride	94.20 ± 4.82	248.60 ± 24.12	170.00 ± 7.45	152.40 ± 7.38	226.98	<0.001 **	P1 < 0.001 ** P2 < 0.001 ** P3 < 0.001 ** P4 < 0.001 ** P5 < 0.001 ** P6 = 0.06
LDL	89.00 ± 7.42	232. ± 22.50	116 ± 12.20	106 ± 12.20	197.38	<0.001 **	P1 < 0.001 ** P2 < 0.001 ** P3 = 0.14 P4 < 0.001 ** P5 < 0.001 ** P6 = 0.136
HDL	55 ± 7.45	14.40 ± 4.55	40 ± 7.45	48 ± 5.73	76.30	<0.001 **	P1 < 0.001 ** P2 < 0.001 ** P3 = 0.020 P4 < 0.001 ** P5 < 0.001 ** P6 = 0.008 *

Data are expressed as mean ± SD; ANOVA test (analysis of variance). LSD (least significant difference); ** highly significant (*p* ≤ 0.001); * significant; P1 = Control group vs. dyslipidemic group; P2 = Control group vs. simvastatin-treated group; P3 = Control group vs. *Spirulina platensis*-treated group; P4 = Dyslipidemic group vs. simvastatin-treated group; P5 = Dyslipidemic group vs. *Spirulina platensis*-treated group; P6 = Simvastatin-treated group vs. *Spirulina platensis*-treated group.

**Table 6:** Comparing MDA nmol/g tissue in the studied groups using ANOVA test.

Group	(Control Group)	(Dyslipidemic Group)	(Simvastatin-Treated Group)	(*Spirulina platensis*-Treated Group)	F	*p* Value
	Mean ± SD of MDA Tissue Level	Mean ± SD of MDA Tissue Level	Mean ± SD of MDA Tissue Level	Mean ± SD of MDA Tissue Level		
LSD	295.6 ± 24.06	375.2 ± 13.9	594 ± 10.91	307 ± 14.6	704.0	<0.001 **
	(Control group)	<0.001 **	<0.001 **	0.131		
	(Dyslipidemic group)		<0.001 **	0.001 **		
	(Simvastatin-treated group)			<0.001 **		

Data are expressed as mean ± SD; one-way ANOVA test (analysis of variance); LSD (least significant difference); ** highly significant (*p* ≤ 0.001).

**Table 7:** Comparing collagen area % in the studied groups using ANOVA test.

Group	(Control Group)	**(Dyslipidemic Group)**	**(Simvastatin-Treated Group)**	**(*Spirulina platensis*-Treated Group)**	F	*p* Value
	Mean ± SD of Collagen Area %	Mean ± SD of Collagen Area %	Mean ± SD of Collagen Area %	Mean ± SD of Collagen Area %		
Post hoc	0.66 ± 0.21	1.45 ± 0.46	8.89 ± 2.81	1.09 ± 0.34	167.9	<0.001 **
	(Control group)	<0.001 **	<0.001 **	0.123		
	Dyslipidemic group		<0.001 **	<0.001 **		
	(Simvastatin-treated group)			<0.001 **		

Data are expressed as mean ± SD; ANOVA test (analysis of variance), LSD (least significant difference); ** highly significant.

**Table 8:** Comparing area % of Bcl2 in the studied groups using ANOVA test.

Group	(Control Group)	(Dyslipidemic Group)	(Simvastatin-Treated Group)	(*Spirulina platensis*-Treated Group)	F	*p* Value ANOVA
	Mean ± SD of Bcl2 Area %	Mean ± SD of Bcl2 Area %	Mean ± SD of Bcl2 Area %	Mean ± SD of Bcl2 Area %		
LSD	41.14 ± 4.26	18.32 ± 4.1	4.99 ± 1.45	38.95 ± 2.32	279.04	<0.001 **
	Control group	<0.001 **	<0.001 **	0.144		
	(Dyslipidemic group)		<0.001 **	<0.001 **		
	(Simvastatin-treated group)			<0.001 **		

Data are expressed as mean ± SD; ANOVA test (analysis of variance), LSD (least significant difference); ** highly significant (*p* ≤ 0.001).

**Table 9:** Comparing the number of TGFβ1 immunopositive cells in the studied groups using ANOVA test.

Group	(Control Group)	(Dyslipidemic Group)	(Simvastatin-Treated Group)	(*Spirulina platensis*-Treated Group)	F	*p* Value
	Mean ± SD of **Number of** TGFβ1 Immunopositive Cells	Mean ± SD of **Number of** TGFβ1 Immunopositive Cells	Mean ± SD of **Number of** TGFβ1 Immunopositive Cells	Mean ± SD of **Number of** TGFβ1 Immunopositive Cells		
LSD	4.60 ± 0.516	22.00 ± 2.404	133.70 ± 30.862	7.20 ± 1.751	158.334	<0.001 **
	Control group	0.017 *	<0.001 **	0.710		
	(Dyslipidemic group)		<0.001 **	0.040 *		
	(Simvastatin-treated group)			<0.001 **		

ANOVA test (analysis of variance), LSD (least significant difference), * significant (*p* ≤ 0.05), ** highly significant (*p* ≤ 0.001).

## Data Availability

The datasets generated and/or analyzed during the current study are available from the corresponding author on reasonable request.
